# Paraneoplastic scleroderma in Kaposi's sarcoma: Report of two cases

**DOI:** 10.1002/ski2.189

**Published:** 2023-02-07

**Authors:** Sara Oulad Ali, Jihane Belcadi, Kaoutar Znati, Marieme Meziane, Nadia Ismaili, Laila Benzekri, Karima Senouci

**Affiliations:** ^1^ Department of Dermatology Mohammed V University in Rabat Ibn Sina University Hospital Rabat Morocco; ^2^ Department of Histopathology Mohammed V University in Rabat Ibn Sina University Hospital Rabat Morocco

## Abstract

Kaposi's sarcoma (KS) is a proliferative and multifocal disease with a double vascular and fibroblastic cell component, of mucocutaneous and visceral expression. It is a multifocal tumoral process, hyperplastic in nature without metastatic potential, induced by the human herpes virus 8 (HHV8). Paraneoplastic syndromes (PS) in KS are rare, with only a small number of cases reported and we have found no previous descriptions of a paraneoplastic scleroderma in KS in the literature. We report the cases of two patients with this atypical PS.

## Graphical abstract

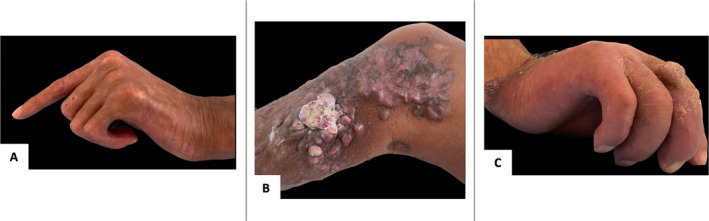

Paraneoplastic syndromes (PS) in Kaposi Sarcoma are rare, with only a small number of cases reported and we have found no previous descriptions of a paraneoplastic scleroderma in KS in the literature. We report the cases of two patients with this atypical PS.

## 1 INTRODUCTION

Kaposi's sarcoma (KS) is a proliferative and multifocal disease with a double vascular and fibroblastic cell component, of mucocutaneous and visceral expression. It is a multifocal tumoral process, hyperplastic in nature without metastatic potential, induced by the human herpes virus 8 (HHV8).

A paraneoplastic syndrome (PS) is a syndrome that is the consequence of neoplasia in the body, due to the production of chemical signalling molecules (such as hormones, chemokines, or cytokines) by tumour cells or by an immune response against the tumour. Unlike a mass effect, it is not due to the local presence of cancer cells.^1^

PS in KS are rare, with only a small number of cases reported and we have found no previous descriptions of a paraneoplastic scleroderma in KS in the literature.

We report the cases of two patients with this atypical PS.

## 2 REPORT OF CASES

### 2.1 Patient 1

**FIGURE 1:**
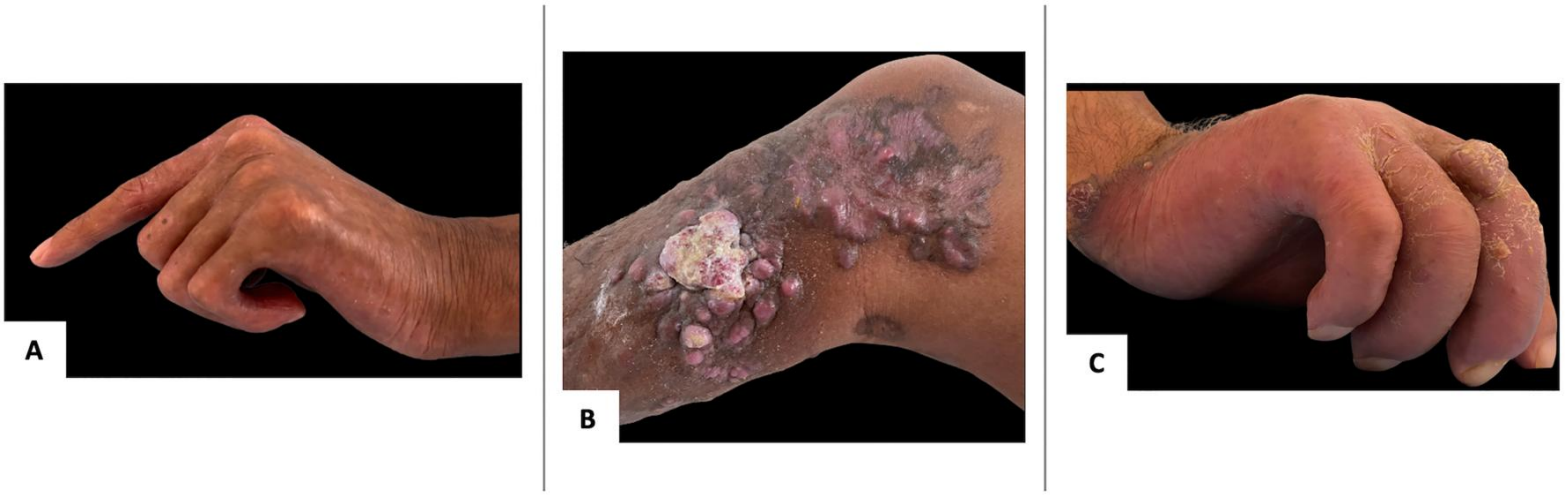
(a) Patient 1. Sclerodactyly of the left hand with irreducible flexion of the last three fingers; (b) Patient 1. Angiomatous nodules of the lower limbs; (c) Patient 2. Sclerodermiform appearance of the right hand

An HIV‐negative 45‐year‐old Moroccan patient, with a history of pulmonary tuberculosis treated in 2020 presented with angiomatous nodules of the lower limbs with a scleroderma‐like condition that has been developing for 12 years. On clinical examination, we found ecchymotic purple nodules of hard consistency based on sclerotic plaques on the lower limbs, with sclerodactyly on the left side (Figure 1a,b). Raynaud phenomenon was absent and no capillaroscopy changes were noted.

The clinical differential diagnosis of the sclerodactyly included systemic sclerosis, acrodermatitis chronica atrophicans, POEMS syndrome and a diabetic cheiroarthropathy.

To rule out this diagnosis, a complete biologic work up was performed, including antinuclear antibodies (ANA) and borreliosis serology which were negative, and a protein electrophoresis which was normal. The electroneuromygram was normal.

Since the skin involvement was quite severe, extensive and disabling, the negativity of the HIV‐test was recontroled twice and also an immune workup was performed to look for an immune deficiency: Laboratory investigations on admission showed normal haemoglobin 13.3 g/dl (normal range, 13–16.5 g/dl), and white blood cell count 9.05 × 10^9^/L (normal range, 4–10 × 10^9^/L). Immunoglobulin assay showed normal IgA 1.6 g/L (normal range, 0.63‐4.84 g/L), IgG 8.88 g/L (normal range, 5.40‐18.22 g/L), and IgM 0.8 g/L (normal range, 0.22‐2.40). The CD4 count was 503 cells/mm^3^ with a CD4/CD8 ratio of 1.2 and CD4% of 34.4%.

**FIGURE 2:**
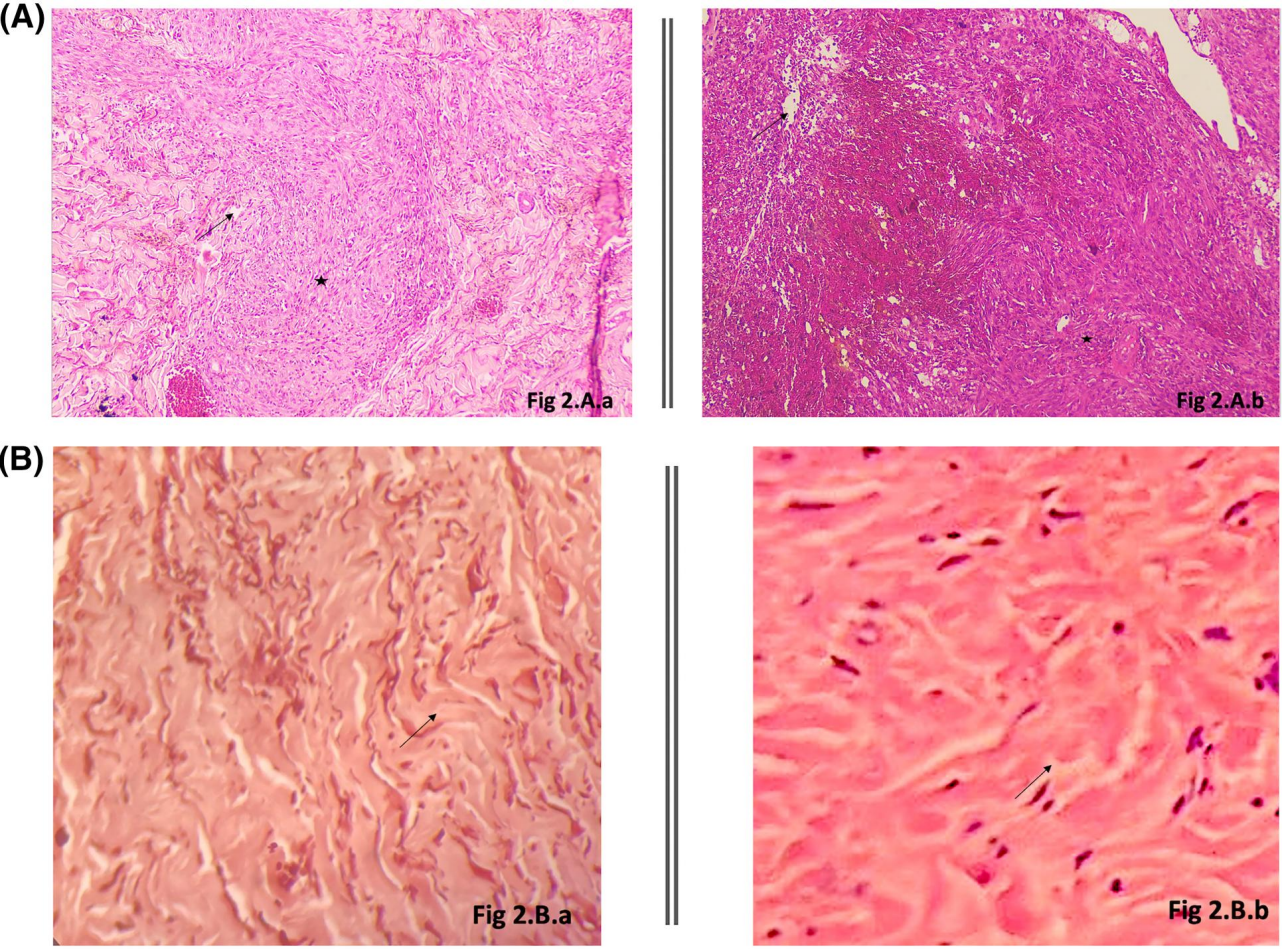
(A) Histologic image of Kaposi Sarcoma: spindle cells (black stars) with vascular slits (black arrows) and vascular structures with a predominance of endothelial cells. (a) Patient 1. (b) Patient 2; (B) Histologic image of sclerodema: the dermis is dense with thick collagen bundles (black arrows) enclosing sweat glands. A rarefaction of vessels in the superficial dermis is noted. (a) Patient 1. (b) Patient 2

Histological examination of the nodules showed spindle cells (Figure 2A.a) expressing the CD34 antigen with a positive HHV8 staining confirming KS, while the histology of the sclerotic lesions on the left hand showed scleroderma (Figure 2B.a).

The extension assessment was carried out including a thoracic‐abdominal‐pelvic computed tomography scan and a fibrocolonoscopy which found no visceral involvement of KS nor systemic sclerosis.

The patient was treated with ABV chemotherapy (doxorubicin, bleomycin and vincristine) due to extensive skin involvement. The evolution was marked by the worsening of the lesions motivating a therapeutic switch to paclitaxel and the patient died after three sessions of taxanes after a septic shock secondary to chemotherapy‐induced immune deficiency.

### 2.2 Patient 2

An HIV‐negative 50‐year‐old Moroccan patient previously healthy, presented with angiomatous nodular lesions localized on all four extremities, which had been developing for 2 years. Clinical examination also found lesions in favour of KS with a sclerodermiform appearance of the right hand characterized by irreducible flexion of the last three fingers (Figure 1c). In this case also, no Raynaud phenomenon nor capillaroscopy changes were noted.

Given the similar differential diagnosis as patient 1, the same biological and radiological assessment were performed eliminating the abovementioned diagnoses and no visceral involvement of KS or systemic sclerosis were found.

A skin biopsy of the nodules confirmed KS (Figure 2A.b) and the histopathological results of the sclerotic lesions showed scleroderma (Figure 2B.b).

Bleomycin‐based monochemotherapy was started for this patient, complicated by febrile pancytopenia, prompting the therapeutic switch to paclitaxel associated with growth factors.

The evolution was marked by the improvement of KS lesions and incomplete regression of sclerodactyly.

## 3 DISCUSSION

KS is a multifocal disease. Its evolutionary spectrum ranges from an indolent locoregional form to a disseminated and fulminant form. PS in KS are rare, with only a small number of cases reported; autoimmune haemolytic anaemia is the most frequent^2^ and three other PS have been reported and are represented in Table 1.^3^, ^4^, ^5^

**TABLE 1:** Literature review of reports on patients with PS in KS other than autoimmune hemolityc anaemia

Reference	Age (years)/sex	Paraneoplastic syndrome	Treatment	Outcome
3	73/M	Dermatomyositis (DM)	Doxorubicine	No relapse of DM after KS treatment
4	62/M	Myasthenia Gravis (MG)	Vinblastine	Improvement of both KS and MG
5	70/M	Paraneoplastic Autoimmune Multi‐organ Syndrome	Vincristine cyclophosphamide, doxorubicin	Died 3 days later

Dermatological paraneoplastic syndromes are a group of cutaneous or mucocutaneous syndromes characterized by their association with neoplasia and by a parallel evolution to the tumour^6^ as in the case of our patients in whom the evolution of the KS and scleroderma was parallel.

The elimination of other diagnoses that could induce a scleroderma‐like disorders helped us to retain the diagnosis of paraneoplastic scleroderma.

Unfortunately in the first case, the patient died due to complications of chemotherapy, without having had time to see the evolution of the sclerotic lesions or the KS.

For the second case, the evolution after treatment was marked by an incomplete resolution of the sclerodactyly. The improvement would be due to the treatment of the KS, which reinforces the theory of paraneoplastic scleroderma.

The incomplete resolution of sclerodactyly is probably due to the importance of the fibrosis and its late stage.

The association between KS and a scleroderma is unclear, we suggest that since in KS both fibroblasts and endothelial cells are infected by HHV8, inducing the production of pro‐angiogenic and inflammatory cytokines,^7^ it could lead to cellular dysfunction and the overproduction of Transforming Growth Factor Beta and Vascular Endothelial Growth Factor that stimulate the fibroblasts to synthesis excess of collagen and fibrosis as it occurs in systemic scleroderma.^8^

Summing up, our case suggests the possibility of the development of scleroderma through paraneoplastic mechanisms in subjects affected with KS. Future studies to shed light on this theory are needed.

## CONFLICT OF INTEREST

The authors have no conflicts of interest to disclose.

## AUTHOR CONTRIBUTIONS

**Sara Oulad Ali**: Conceptualization (Equal); Data curation (Equal); Formal analysis (Equal); Investigation (Equal); Methodology (Equal); Project administration (Equal); Resources (Equal); Software (Equal); Supervision (Equal); Validation (Equal); Visualization (Equal); Writing – original draft (Lead); Writing – review & editing (Lead). **Belcadi Jihane**: Conceptualization (Equal); Data curation (Equal); Formal analysis (Equal); Investigation (Equal); Methodology (Equal); Project administration (Equal); Resources (Equal); Software (Equal); Supervision (Equal); Validation (Equal); Visualization (Equal); Writing – original draft (Supporting); Writing – review & editing (Supporting). **Kawtar Znati**: Conceptualization (Equal); Data curation (Equal); Formal analysis (Equal); (Equal); Investigation (Equal); Methodology (Equal); Project administration (Equal); Resources (Equal); Software (Equal); Supervision (Equal); Validation (Equal); Visualization (Equal); Writing – original draft (Supporting); Writing – review & editing (Supporting); Mariame Meziane: Conceptualization (Equal); Data curation (Equal); Formal analysis (Equal); Investigation (Equal); Methodology (Equal); Project administration (Equal); Resources (Equal); Software (Equal); Supervision (Equal); Validation (Equal); Visualization (Equal); Writing – original draft (Supporting); Writing – review & editing (Supporting). **Nadia Ismaili**: Conceptualization (Equal); Data curation (Equal); Formal analysis (Equal); Investigation (Equal); Methodology (Equal); Project administration (Equal); Resources (Equal); Software (Equal); Supervision (Equal); Validation (Equal); Visualization (Equal); Writing – original draft (Supporting); Writing – review & editing (Supporting). **Laila Benzekri**: Conceptualization (Equal); Data curation (Equal); Formal analysis (Equal); Investigation (Equal); Methodology (Equal); Project administration (Equal); Resources (Equal); Software (Equal); Supervision (Equal); Validation (Equal); Visualization (Equal); Writing – original draft (Supporting); Writing – review & editing (Supporting). **Karima Senouci**: Conceptualization (Equal); Data curation (Equal); Formal analysis (Equal); Investigation (Equal); Methodology (Equal); Project administration (Equal); Resources (Equal); Software (Equal); Supervision (Equal); Validation (Equal); Visualization (Equal); Writing – original draft (Supporting); Writing – review & editing (Supporting).

## ETHICS STATEMENT

The patients' caregivers in this manuscript have given written informed consent to the publication of their case details.

## Data Availability

Data sharing is not applicable to this article as no new data were created or analyzed in this study.

## References

[1] Darnell RB , Darnell R , Posner JB . Paraneoplastic syndromes. Oxford: Oxford University Press; 2011.

[2] Puthenparambil J , Lechner K , Kornek G . Autoimmune hemolytic anemia as a paraneoplastic phenomenon in solid tumors: a critical analysis of 52 cases reported in the literature. Wien Klin Wochenschr. 2010;122(7‐8):229–36. 10.1007/s00508-010-1319-z 20503022

[3] Alghanim K , Gasmelseed B . Kaposi’s sarcoma associated with adult dermatomyositis. Saudi Med J. 2021;42(5):570–3. 10.15537/smj.2021.42.5.20200583 33896788 PMC9149691

[4] Mantero V , Mascolo M , Bandettini di Poggio M , Caponnetto C , Pardini M . Myasthenia gravis developing in an HIV‐negative patient with Kaposi’s sarcoma. Neurol Sci. 2013;34(7):1249–50. 10.1007/s10072-012-1201-z 23010878

[5] Ghigliotti G , Di Zenzo G , Rongioletti F , Col E , Pastorino C , et al.et al. Paraneoplastic autoimmune multi‐organ syndrome: association with retroperitoneal Kaposi’s Sarcoma. Acta Derm Venereol. 2016;96(2):261–2. 10.2340/00015555-2169 26072713

[6] Saurat J‐H , Lipsker D , Thomas L , Borradori L , Lachapelle J‐M . Dermatologie et infections sexuellement transmissibles. 6^th^ ed; 2017.

[7] Gramolelli S , Ojalal P . Kaposis sarcoma herpesvirus‐induced endothelial cell reprogramming supports viral persistence and contributes to Kaposis sarcoma tumorigenesis. Curr Opin Virol. 2017;26:156–62. 10.1016/j.coviro.2017.09.002 29031103

[8] Didier K , Robbins A , Antonicelli F , Pham B , Giusti D , Servettaz A . Updates in systemic sclerosis pathogenesis: toward new therapeutic opportunities. La Revue de Médecine Interne. 2019;40(October):654–63. 10.1016/j.revmed.2019.05.016 31301944

